# Scalable point cloud meshing for image-based large-scale 3D modeling

**DOI:** 10.1186/s42492-019-0020-y

**Published:** 2019-08-07

**Authors:** Jiali Han, Shuhan Shen

**Affiliations:** 1 National Laboratory of Pattern Recognition, Institute of Automation, Chinese Academy of Sciences, Beijing, 100190 China; 2 University of Chinese Academy of Sciences, Beijing, 100049 China

**Keywords:** Mesh-generation, Delaunay-based optimization, Large-scale scenes

## Abstract

Image-based 3D modeling is an effective method for reconstructing large-scale scenes, especially city-level scenarios. In the image-based modeling pipeline, obtaining a watertight mesh model from a noisy multi-view stereo point cloud is a key step toward ensuring model quality. However, some state-of-the-art methods rely on the global Delaunay-based optimization formed by all the points and cameras; thus, they encounter scaling problems when dealing with large scenes. To circumvent these limitations, this study proposes a scalable point-cloud meshing approach to aid the reconstruction of city-scale scenes with minimal time consumption and memory usage. Firstly, the entire scene is divided along the *x* and *y* axes into several overlapping chunks so that each chunk can satisfy the memory limit. Then, the Delaunay-based optimization is performed to extract meshes for each chunk in parallel. Finally, the local meshes are merged together by resolving local inconsistencies in the overlapping areas between the chunks. We test the proposed method on three city-scale scenes with hundreds of millions of points and thousands of images, and demonstrate its scalability, accuracy, and completeness, compared with the state-of-the-art methods.

## Introduction

3D modeling of large-scale scenes has attracted extensive attention in recent years. It can be applied in many ways such as virtual reality, urban reconstruction, and cultural heritage protection. Nowadays, there are many techniques for obtaining the point cloud of large scenes; the laser-scanner-based and image-based methods appear to be the most widely used. Terrestrial laser scanners can efficiently obtain billions of points [1–3]. The image-based method takes multi-view images as the input, and produce per-pixel dense point clouds using the structure-from-motion (SfM) and multi-view stereo (MVS) algorithms [4–7]. For city-scale scene reconstructions, the image-based modeling approach is more convenient and cost-effective, because of the rapid developments of drones and oblique photography. However, noise and outliers are unavoidably included in the MVS point cloud. Thus, extracting a watertight mesh model from noisy MVS point clouds is a key step toward ensuring the 3D model’s quality.

Surface reconstruction from point clouds has extensively been researched in the field of computer graphics, and there are various reconstruction methods in terms of the input point clouds. The Poisson surface reconstruction (PSR) (such as refs. [8–10]) is a popular point meshing algorithm. It frames the surface reconstruction as a spatial Poisson problem, defines an indicator function to represent the surface model, and uses the points and estimated normal vectors to obtain the solution of the function by solving the Poisson equation. Finally, the approximate surface model with the entity information is obtained by extracting the isosurface directly. The PSR is a global optimization method, and the reconstructed mesh model based on it is watertight, with detailed characteristics. Another traditional surface reconstruction method is marching cubes [11], which uses the divide-and-conquer strategy. It fits the surface into a cube, and processes all the cubes sequentially. For each cube, the surface intersections are identified via linear interpolation, and the inner isosurface is approximated by triangles. Finally, a polygonal mesh can be extracted. There are many variations of this method (such as refs. [12–15]).

In addition, there are several image-based methods for surface reconstruction. One of the most important methods is based on the Delaunay triangulation [16], a global optimization algorithm and the basis for several other methods (such as refs. [5, 17–25]). This approach considers the inevitable noise and outliers in the MVS point cloud and exploits the visibility information of cameras, thereby producing a better surface than the traditional approaches. As a state-of-the-art algorithm in image-based surface reconstruction, ref. [5] defines surface reconstruction as a global optimization problem, and obtains a complete result. However, as the size of the point cloud increases, problem-solving will consume so much time and memory that impedes the efficiency of the computer. This study aims to solve this challenge.

When applied to large-scale point clouds, the traditional approaches encounter bottlenecks due to the drastic increase in time and memory. Some studies have been conducted to address these problems. Using the marching cubes and the results obtained in ref. [26], Wiemann et al. [27] handled large-scale data using an octree-based optimized data structure and a message-passing-interface (MPI)-based distributed normal estimation provided by the Las Vegas surface reconstruction toolkit that can assign the data to a computing cluster [28]. They incorporated parallelization into it, and proposed a grid-extrusion method to replace the missing triangles by adding new cells dynamically. Subsequently, Wiemann et al. [2] used a collision-free hash function in place of the octree structure to manage the voxels in a hash map to obtain better results. This function can instantaneously identify the adjacent cells under certain conditions. Wiemann et al. [2], when handling the data, serialized them into chunks that are geometrically related; then, the partitions are sent to the slave nodes to be rebuilt in parallel. However, this method may have the undesirable effect of generating more triangles than necessary in the mesh.

Gopi et al. [29] proposed an unique and fast project-based method to incrementally develop an interpolatory surface. Although, their approach has linear-time performance, it cannot effectively handle the sharp curvature variation. Marton et al. [30] circumvented some of the challenges [29] cannot handle. Their method is based on incremental surface growing [31], an approach that does not require interpolation, and can preserve all the points. However, their approach is a greedy type, and it is not guaranteed to obtain the same result as the global optimal solution. More recently, Er et al. [32] proposed a new approach whereby the data is sampled and reconstructed based on the witness complex method [33], using the original data as the constraint. After sampling, although the size of the data may be smaller, it is difficult to approximate the sampling rate for the different datasets, which affects the final reconstruction result. Ummenhofer and Brox [34] and Fuhrmann and Goesele [35] have also researched large-scale reconstructions. The former proposed a global energy cost function, and they extracted the surface by conducting energy minimization on a balanced octree. However, this method does not solve the scale problem that characterizes large-scale reconstruction due to its global formulation. The latter proposed a local approach that is parameter-free for datasets, and applicable to large, redundant, and potentially noisy point clouds. However, this local approach will generate many gaps, and cannot fill larger holes. Recently, Mostegel et al. [36] proposed a scalable approach that can process enormous point clouds. They used an octree to divide the data, and run meshing method locally. The final surface can be obtained by extracting overlaps and filling the holes with a graph-cut algorithm. This method is able to obtain a watertight mesh for extremely large point clouds. However, the octree structure necessitates several repetition of the calculation to obtain enough overlaps, thereby increasing time consumption and memory usage.

**Fig. 1:**
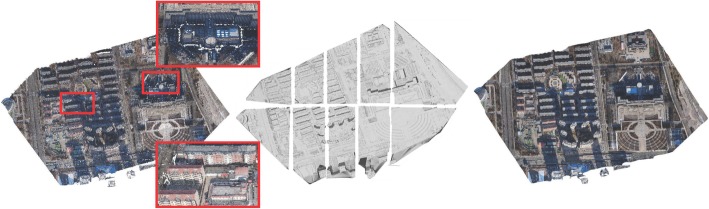
City-scale scene surface modeling using the proposed scalable point-cloud-meshing method. The left is the input point cloud and two enlarged building areas. The middle is the result of incorporating region partitioning into local meshes, and the right is the final merged mesh with texture

To circumvent the limitations of current state-of-the-art methods, we propose a scalable point-cloud meshing approach that can efficiently process city-scale scenes based on MVS points with minimal memory usage. As shown in Fig. 1, we first divide the entire scene into several chunks with overlapping boundaries along the *x* and *y* axes, following which we perform the Delaunay-based optimization to extract the mesh for each chunk in parallel. Finally, the local meshes are merged by resolving the inconsistencies in the overlapping areas between the chunks. The main contributions of this study are as follows:

- We propose a practicable and efficient scalable meshing approach to handling MVS points with minimal and adjustable memory that can obtain a reconstructed surface similar to that generated by the global-based method [5].
- We achieve a region-partition method that can divide the scene into chunks with overlapping boundaries, each chunk being compatible with the computer memory. In this method, each overlapping grid is calculated two or four times, thus eliminating some redundant computations in ref. [36].

## Methods

In this study, we deal with a large-scale point cloud computed from images that are generated using the SfM and MVS algorithms. Without any auxiliary sensor information, the point cloud generated from the SfM and MVS lies in an arbitrary coordinate. However, for outdoor scenes, it can easily be transformed according to the geographical coordinates using the camera’s in-built GPS information, or using the ground control points for greater precision. This study mainly focuses on outdoor city-scale scenes; thus, we assume that the MVS point cloud has already been geo-referenced. Therefore, it is reasonable to partition the scene on the ground plane (*x*-*y* plane), but not along the vertical axis (*z*-axis).

**Fig. 2:**
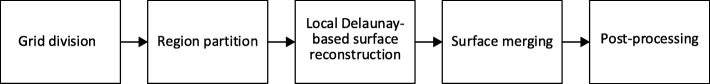
The pipeline of the proposed scalable point-cloud-meshing method

The pipeline of the proposed method is shown in Fig. 2; it has three main steps: region partition, local surface reconstruction using Delaunay-based optimization, and surface merging. All three steps are detailed in the following subsections.

### Region partitioning

Region partitioning is a straightforward strategy for solving memory limitation problems by partitioning large point clouds into chunks, and processing each one individually before merging them. Our point-cloud partitioning process incorporates the region partitioning into the approach of Mostegel et al. [36]. In ref. [36], they divide the point cloud into voxels managed by an octree structure, and run local computation on all the voxel subsets to extract the surface hypotheses. However, their process inevitably results in the repetition of many facets. Consequently, for large-scale scenes, there will be several voxels, and the computation on a voxel will be repeated many times, leading to redundancy. To circumvent this limitation, we propose region partitioning.

**Fig. 3:**
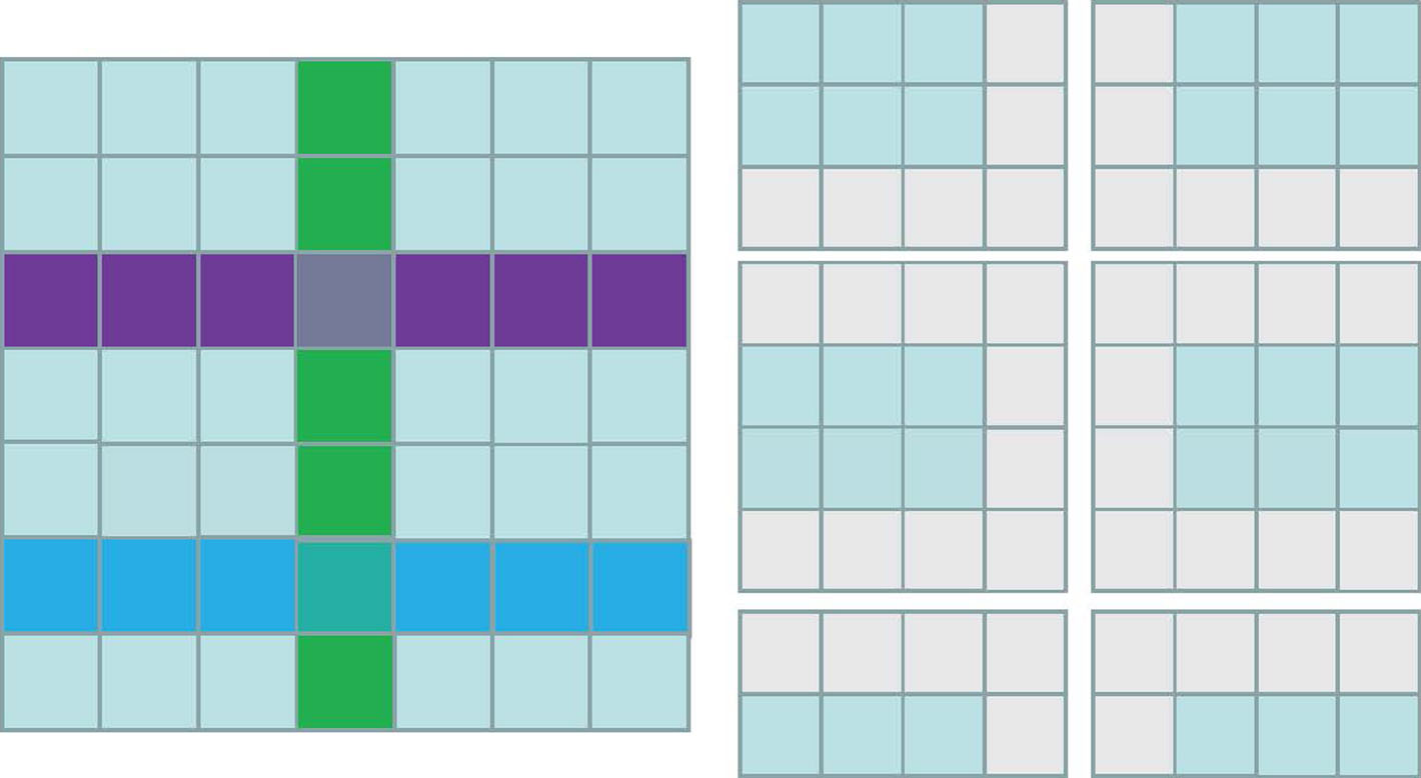
An example of region partition. We mark the extracted grids with different colors (the left) that will be incorporated into their adjacent parts as the boundaries. The order of grid extraction is green, purple, and blue; in the end, the scene is divided into several overlapping chunks (the right)

We first divide the point cloud into regular grids on the *x-y* plane; each grid contains all the points whose *x* and *y* coordinates are within it. The grid is treated as the smallest unit. Given the maximum number of points that a single computer node can handle, the challenge of partitioning the grids accordingly into chunks arises. Here, we extract the grids along the *x* and *y* axes, and the scene can be divided into portions. The extracted grids will be incorporated into their adjacent parts as boundaries; extraction will be performed repeatedly and adjusted until the number of points in each part falls below the maximum we set (designated *N*_*max*_). Finally, we can obtain a group of chunks with overlapping boundaries and limited number of points that will be processed in parallel (Fig. 3).

### Delaunay triangulation and minimum s-t cut

We run the Delaunay-based optimization algorithm locally on each chunk to obtain the local surfaces, after which the local mesh is cleaned to obtain more consistent local surfaces.

#### Local Delaunay-based surface computation

The local surface-reconstruction algorithm is based on the method by Vu et al. [5]. First, the Delaunay triangulation is performed using the point cloud. Then, the visibility of the points and the quality of the surface are used to build the energy function representing the energy for extracting the final surface that can be obtained using the global minimizing function with the minimum s-t cut algorithm. The energy function is defined as follows:

1 $$ E(S)= Evis(S)+\lambda \cdotp Equal(S) $$

where *S* is the final surface and *λ* is a balance factor. In this study, we use *λ* = 0.5, which can achieve favorable results across all the experiments; this value is also the default setting in OpenMVS [37].

In Eq. 1, the visibility term *Evis(S)* conforms to the principle that the line of sight from the cameras to the points should not cross the final surface. Thus, it fully exploits the visibility of points, and can effectively filter out outliers. Besides, the quality item *Equal(S)* is defined to penalize triangles with improper size or edge length, both of which tend to have less visibility than the others on dense surfaces. The evaluation criterion of the triangles is related to the angle between a triangle and its empty circumspheres.

Following the minimum s-t cutting, every tetrahedron is labeled as inside or outside; the triangles that lay between both constitute the surface. Note that not all the points are inserted during Delaunay triangulation. A point can only be inserted when the re-projection distance between it and the other points that have been inserted exceeds a certain distance [38] that is set to be compatible with the computer memory and, effectively reconstruct the places with overly dense points.

#### Local mesh clean up

Some extent of cleaning is performed to eliminate noise after local surface reconstruction for each chunk, which includes removing non-manifold and overly long edges, isolating components and vertexes connected to a single facet or none, and filling the holes. The cleaning process is necessary because the Delaunay-based method cannot obtain the most complete and consistent surface at once; furthermore, cleaning is not as time-consuming as the other steps. It may be observed that hole filling is necessary, because some facets may be removed in the process of removing edges that are too long. The hole-filling algorithm here is implemented by the Visualization and Computer Graphics Library (VCG) [39] and is a heuristic algorithm that can fill holes with the specified side length as far as possible.

### Surface merging

Once the local surfaces for each chunk are generated, using the overlapping boundaries as an intermediate, we merge them by extracting proper facets, and resolving the inconsistencies in the overlapping areas.

#### Consistent triangle extraction

The local surfaces are computed individually, and inconsistent facets exist mainly in the boundary grids. To resolve these inconsistencies, we first extract the triangles located in the internal grids (not boundary grids) that are computed just once. Then, we extract the triangles that span the internal and boundary grids, because in the areas between the internal and boundary grids, these triangles are farther from the outer boundaries of the chunk than the triangles generated by the other chunks, and the Delaunay tetrahedralization will be more stable. These triangles are more suitable for selection and are consistent with the first kind of triangles extracted.

Following this, we focus on extracting the triangles within the boundaries, and they are computed two or four times. We extract repeated triangles that are repeated the same number of times as the grids where they are located. These repeated triangles are also consistent, because they are the same in all adjacent chunks. Based on the efficiency of the Delaunay-based method, most triangles in the boundaries are repeated ones, thus, simplifying the subsequent hole-filling task to an extent.

#### Hole filling

By combining the selected consistent triangles above, we can obtain a surface mesh with some holes in the boundary grids. Then, to minimize these inconsistencies, we attempt to fill these holes. This step is similar to the method in ref. [36]. We remove triangles that will intersect the surface or generate non-manifold edges if they are added first, and then cluster the rest by the edge connectivity in each chunk. Specifically, we put the triangles that can merge to form only one connected domain in a chunk, and refer to each group of triangles after clustering as a *patch*. The *patch* will be used as the smallest unit for hole filling. It is better to prioritize patches that are farther from the outer boundaries of a chunk, because Delaunay tetrahedralization is more stable in these regions, compared to those close to the outer boundaries. We use the centroid of a *patch* to represent the average position of all the points in the *patch,* and find the outer boundaries of the chunk where the *patch* is located. The farther the centroid is from these outer boundaries, the higher its selection priority. We define the offset of a *patch* P as follows:

2 $$ offset\left(\mathrm{P}\right)=\min \left(\underset{b_x\in {B}_x}{\min}\left(\left|{c}_x-{b}_x\right|\right),\underset{b_y\in {B}_y}{\min}\left(\left|{c}_y-{b}_y\right|\right)\right) $$

Where *c*_*x*_ and *c*_*y*_ are the *x* and *y* coordinates of the centroid of P and *B*_*x*_ and *B*_*y*_ are the *x-*and-*y-*coordinate sets of the outer boundaries of the chunk where P is located.

We sort the patches by their offset in descending order and visit them sequentially. If a *patch* does not cross the surface and generate non-manifold edges, it will be added to the final surface. Note that patches are used instead of single triangles for hole filling because using the former can reduce the number of required checks (checks for intersections or generation of non-manifold edges). This step can effectively fill the holes caused by inconsistent boundary computation.

Finally, we remove the non-manifold vertexes using VCG [39] and apply HC-Laplacian smoothing [40] as a post-processing step to obtain a smoother surface. These tasks were not performed when the local meshes were being cleaned, because they displace the points and change the topology of meshes, thereby greatly reducing the number of repeated facets in boundaries. Note that we cannot theoretically guarantee that the final result has no holes (likewise in the global optimization based method [5]), but from the experimental results, most areas of the surface are watertight; occasionally, there may be few small holes where noise is particularly large. Besides, when the input point cloud contains isolated outliers somewhere (for example, for the aerial photography of urban scenes, some outliers may appear deep below the ground plane, which although being very rare cannot be completely ruled out), we may incorporate them into our final surface. However, this problem is not difficult to resolve. We can eliminate the noise using the visibility information by finding the visibility of the points in the cameras of points. If a point is not visible in any of the cameras that has acquired it, it can be considered big noise and removed.

## Results and discussion

The proposed method is evaluated by varying the partition numbers, and comparing it with other state-of-the-art approaches. Here, we used a 20-core workstation with 2.4 GHz CPU and 128 GB RAM. The API development environment of our experiments is Ubuntu 18.04, 64 bit.

### Datasets and parameters

**Fig. 4:**
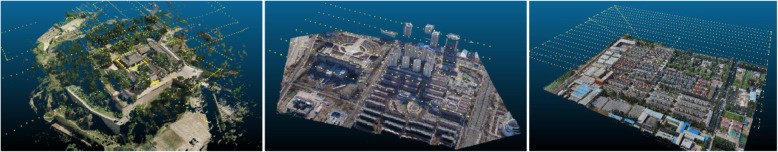
Illustration of the point cloud and cameras of the Temple, City1 and City2 datasets; the yellow marks represent the positions of the cameras

We use three large-scale datasets, Temple, City1, and City2, all obtained using drone aerial photography. For all three datasets, the points are computed from the images using off-the-shelf SfM [41, 42] and MVS [37] algorithms. A detailed description of the datasets is shown in Table 1, and the illustration of the point clouds, as well as the cameras, is shown in Fig. 4.

**Table 1:** The description of our datasets

Dataset	No. of images	No. of points	Area (km^2^)	Scene features
Temple	2854	67 Million	0.06	Ancient Chinese buildings, Forests
City1	930	96 Million	1.68	City buildings, Squares, Roads
City2	7450	126 Million	0.81	Houses, Streets

The proposed algorithm has two main parameters: grid size, *δ*, and maximum number of points in one chunk, *N*_*max*_. *δ* is set according to the size of objects in the scene, and we set *δ* as 6 m for all the datasets. The *N*_*max*_ values are determined by the limits of the computing resources (mainly the memory).

### Evaluation of different partition numbers

**Fig. 5:**
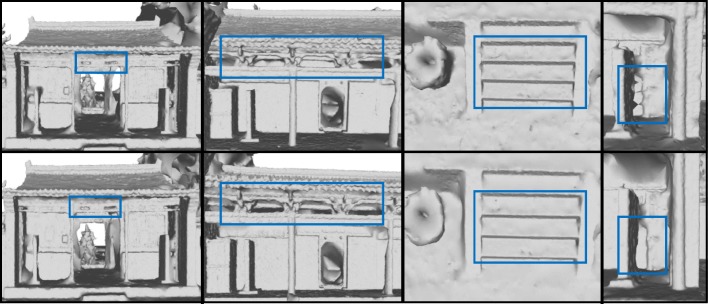
Comparison between *N*_*max*_ = 1.3 × 10^7^ and *N*_*max*_ = 6.0 × 10^6^. The first row is the results for *N*_*max*_ = 6.0 × 10^6^, and the second is for *N*_*max*_ = 1.3 *×* 10^7^. When *N*_*max*_ = 6.0 *×* 10^6^, the quality of merged surface is reduced to some extent, compared with the results obtained when *N*_*max*_ = 1.3 *×* 10^7^ is used in some areas, which reflects the balance between memory consumption and reconstruction completeness

The partition numbers are affected by the upper limit of the number of points in a chunk. To verify the robustness of the proposed algorithm against the number of chunks, we modify *N*_*max*_ from 1.3 *×*10^7^ to 9.0 *×* 10^6^, and 6.0 *×* 10^6^, to vary the number of partitions, and evaluate the results on the Temple dataset. As may be seen in Fig. 5, as the number of points in a chunk decreases, although the reconstruction result is still good, the completeness and accuracy are relatively compromised. With different partition numbers, we record the running time of the main steps in our method and the peak memory consumption, as shown in Table 2. As may be observed, as the *N*_*max*_ decreases, the algorithm consumes less memory, and the local Delaunay-based computation is less time-consuming; however, surface merging (mainly hole filling) becomes more time-consuming. Therefore, our method is more advantageous when the partition numbers are relatively small. We prefer to choose *N*_*max*_ based on the memory limit of the computer, because the number of partitions obtained is directly proportional to the number of holes to be filled and the required computation time.

**Table 2:** Results for different *N*_*max*_

*N*_*max*_(*×* 10^6^)	*T1* (s)	*T2* (s)	*T3* (s)	Peak memory consumption (GB)/per process
13	1408	113	829	5
9	957	140	1409	4
6	809	148	1599	4

*T1* represents the duration of parallel local surface reconstruction, *T2* is the duration of extracting consistent triangles, and *T3* is the duration of hole filling

### Comparison with state-of-the-art approaches

In this part, we qualitatively compare our method with the state-of-the-art methods [5, 34, 35, 43] for the three datasets. First, we compare our method with the global Delaunay-based optimization method [5] (hereafter referred to as “Global”). We also compare our method with the global dense multiscale reconstruction (GDMR) [34, 43] and the floating scale surface reconstruction (FSSR) [35]. These two methods are based on the implicit functions, and instead of visibility information, they deploy a scale parameter that affects the time and memory consumption and reconstruction completeness. FSSR and GDMR are 64-bit executable programs provided respectively by refs. [44, 45]. Global is the codes in OpenMVS [37]. In our method, we use *N*_*max*_ = 1.3 *×* 10^7^; for FSSR and GDMR, we use the same scale parameters according to the length of the edges from one point to its neighbors; thus, for both, we use 0.08 on the Temple dataset, 1.5 on the City1 dataset, and 1.0 on the City2 dataset, which yield the optimal results we try.

#### Completeness

**Fig. 6:**
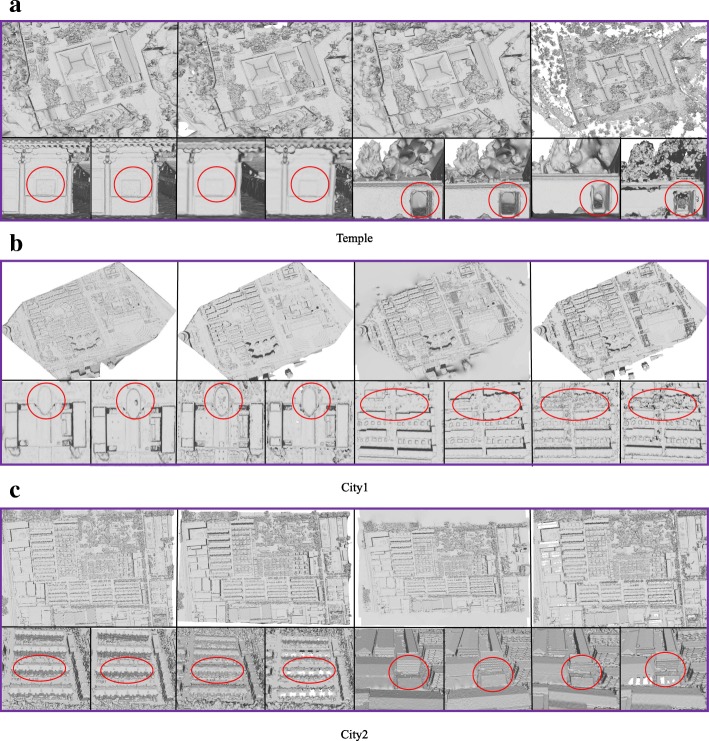
Comparison of the different methods on the Temple, City1, and City2 datasets. In all [(**a**), (**b**) and (**c**)], the top row, from left to right, are the reconstruction results of our proposed method, Global, GDMR, and FSSR, respectively. The next row contains two details of the surface, and the columns in the two details represent the result of our method, Global, GDMR, and FSSR, respectively. The red circles highlight the surface quality of our approach, demonstrating that our proposed method and Global can obtain similar results. Both methods outperform GDMR and FSSR in surface details and completeness

We first compare the reconstruction completeness of these methods. We can see in Fig. 6 that the FSSR does not effectively fill larger holes; Global and our method outperform it in this aspect. Furthermore, Global and our method can also retain more details with less noise than GDMR and FSSR.

#### Time and memory consumption

All the methods are run on the CPU. The local reconstruction work in the proposed method is run in parallel, while that of Global is run sequentially. When we run the executable programs of FSSR and GDMR, we find that parallel computations are added when certain operations are performed. The time consumption and memory usage of the different methods can be seen in Table 3, from which it may be observed that our method outperforms Global in memory usage and time consumption. FSSR and GDMR run faster sometimes because they do not utilize the visibility information of the points and cameras; however, there is a tradeoff between this speed and the capacity to retain details and scene completeness. Thus, in terms of detail retention and scene completeness, our method and Global outperform FSSR and GDMR.

**Table 3:** The different methods and the time (min) and peak memory consumption (GB) for the three datasets

		Global	Proposed method	FSSR	GDMR
Temple	Time	50	40	105	50
	Memory	16	5	10	9
City1	Time	71	39	27	29
	Memory	93	18	6	7
City2	Time	57	32	37	25
	Memory	108	8	8	7

## Conclusions

In this paper, we propose a scalable point-cloud meshing approach to image based 3D modeling that can enable the reconstruction of large-scale scenes with minimal memory usage and time consumption. Different from the current distributed points meshing algorithms [36] based on the regular voxel partition of the scene, we propose a region-partitioning method that can divide a scene into several chunks with overlapping boundaries, each chunk satisfying the memory limit. Then, the Delaunay-based optimization is used to extract the mesh for each chunk in parallel. Finally, local meshes are merged by resolving local inconsistencies on the overlapping areas between the chunks. We evaluate the proposed method on three city-scale scenes with hundreds of millions of points and thousands of images, and demonstrate its scalability, accuracy, and completeness, compared with the state-of-the-art methods.

In this study, the hole-filling task was performed as a sequential computation. However, in future work, we will mainly focus on achieving simultaneous parallel computation when filling the holes to further improve the running speed and efficiency of our method.

## Data Availability

The datasets used and/or analysed during the current study are available from the corresponding author on reasonable request.

## Abbreviations

FSSR: Floating scale surface reconstruction
GDMR: Global dense multiscale reconstruction
MPI: Message-passing-interface
MVS: Multiple view stereo
PSR: Possion surface reconstruction
SfM: Structure-from motion
VCG: Visualization and Computer Graphics Library
